# PRECOGx: e**x**ploring GPCR signaling mechanisms with deep protein representations

**DOI:** 10.1093/nar/gkac426

**Published:** 2022-05-26

**Authors:** Marin Matic, Gurdeep Singh, Francesco Carli, Natalia De Oliveira Rosa, Pasquale Miglionico, Lorenzo Magni, J Silvio Gutkind, Robert B Russell, Asuka Inoue, Francesco Raimondi

**Affiliations:** Laboratorio di Biologia Bio@SNS, Scuola Normale Superiore, Piazza dei Cavalieri 7, 56126, Pisa, Italy; Heidelberg University Biochemistry Centre, 69120 Heidelberg, Germany; BioQuant, Heidelberg University, 69120 Heidelberg, Germany; Laboratorio di Biologia Bio@SNS, Scuola Normale Superiore, Piazza dei Cavalieri 7, 56126, Pisa, Italy; Laboratorio di Biologia Bio@SNS, Scuola Normale Superiore, Piazza dei Cavalieri 7, 56126, Pisa, Italy; Laboratorio di Biologia Bio@SNS, Scuola Normale Superiore, Piazza dei Cavalieri 7, 56126, Pisa, Italy; Laboratorio di Biologia Bio@SNS, Scuola Normale Superiore, Piazza dei Cavalieri 7, 56126, Pisa, Italy; Department of Pharmacology and Moores Cancer Center, University of CA, San Diego, La Jolla, CA 92093, USA; Heidelberg University Biochemistry Centre, 69120 Heidelberg, Germany; BioQuant, Heidelberg University, 69120 Heidelberg, Germany; Graduate School of Pharmaceutical Sciences, Tohoku University, Sendai, Miyagi 980-8578, Japan; Laboratorio di Biologia Bio@SNS, Scuola Normale Superiore, Piazza dei Cavalieri 7, 56126, Pisa, Italy

## Abstract

In this study we show that protein language models can encode structural and functional information of GPCR sequences that can be used to predict their signaling and functional repertoire. We used the ESM1b protein embeddings as features and the binding information known from publicly available studies to develop PRECOGx, a machine learning predictor to *explore* GPCR interactions with G protein and β-arrestin, which we made available through a new webserver (https://precogx.bioinfolab.sns.it/). PRECOGx outperformed its predecessor (e.g. PRECOG) in predicting GPCR-transducer couplings, being also able to consider all GPCR classes. The webserver also provides new functionalities, such as the projection of input sequences on a low-dimensional space describing essential features of the human GPCRome, which is used as a reference to track GPCR variants. Additionally, it allows inspection of the sequence and structural determinants responsible for coupling via the analysis of the most important attention maps used by the models as well as through predicted intramolecular contacts. We demonstrate applications of PRECOGx by predicting the impact of disease variants (ClinVar) and alternative splice forms from healthy tissues (GTEX) of human GPCRs, revealing the power to dissect system biasing mechanisms in both health and disease.

## Graphical Abstract

**Graphical Abstract:**
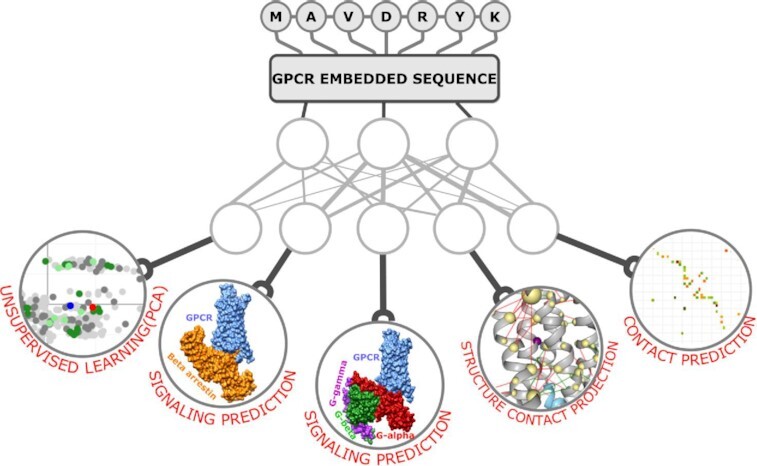
Protein language models can encode structural and functional information of GPCR sequences which is used to predict their signaling and functional mechanisms, such as coupling with G protein and β-arrestin and their sequence and structural determinants. Projection of the input sequence on a low-dimensional space (PCA) describes the essential features of the human GPCRome.

## INTRODUCTION

G protein-coupled receptors (GPCRs) form the largest family of cell-surface receptors and the most important pharmacological class, being targeted by approximately one-third of the marketed drugs (1). They transduce a multitude of physico-chemical stimuli from the extracellular environment to activate intracellular signalling pathways through the coupling to one or more heterotrimeric G proteins, which are grouped into four major G protein families: G_s_, G_i/o_, G_q/11_ and G_12/13_ based on their α-subunits (2). GPCRs’ downstream activity is controlled by β-arrestins, which offer an alternative layer of signalling modulation via ERK (3). Alteration of these transduction mechanisms is linked to a myriad of pathological states (i.e. signalopathies), including cancer (4–7). A deeper knowledge of these mechanisms, integrated in the wider biological context of a disease state, can impact targeted therapies and personalized medicine protocols (e.g. (8)). Dissecting GPCR-G protein coupling can also aid the design of chemogenetic tools, such as Designer Receptors Exclusively Activated by Designer Drugs (DREADDs), that can be of great use in tinkering with signalling pathways in living systems (9). Ligand binding to GPCRs induces conformational changes that lead to binding and activation of G proteins situated intracellularly. Mammalian GPCRs display a wide and distinct repertoire of G protein coupling, ranging from highly selective to promiscuous profiles, which lead to specific downstream cellular responses (6). Determining specific coupling profiles is critical to understanding GPCR biology and pharmacology. Structural determination of receptor/G protein complexes is advancing rapidly, with over 170 complex structures deposited in the PDB (as of March 2022). This unprecedented wealth of structural information is illuminating the basis of receptor activation across classes (10), G protein families (e.g. (11)), as well as among distinct transducers of the same receptor (e.g.(12)). At the same time, quantitative screening methodologies have been set up to systematically profile the binding activities of GPCRs for transducer proteins ((13–16)). Despite these continuous advancements, a consensus picture of the sequence and structural basis of selectivity is still far from being complete and, importantly, coupling information is still missing for many receptors. Approximately 28% of human, non-olfactory GPCRs still lack the coupling information according to either IUPHAR/GuidetoPharmacology (GtoPdb) (17) or quantitative coupling studies, preventing a deeper understanding of their biological function.

To fill this knowledge gap, we previously developed PRECOG (18), a machine learning-based predictor of Class A GPCRs coupling with G proteins. In this previous study, we used sequence- and structure-based features and trained on experimentally determined binding activities of 144 Class A human GPCRs across 11 chimeric G proteins obtained through the TGFα shedding assay (TGF) (13,14). We herein present PRECOGx, a new ML-based predictor of G protein and β-arrestin binding which relies on protein embeddings from a pre-trained protein language model, i.e. the Evolutionary Scale Model (ESM) (19). ESM has been derived from Natural Language Processing (NLP) state-of-art models, i.e. transformers (20), and has shown superior performances in a number of protein structure and function prediction tasks as it captures aminoacids’ contextual dependencies within sequence (19). ESM was shown to outperform competing methods for protein embeddings (e.g. SeqVec (21) or Unirep (22)) and similar architectures, i.e. Evoformer, form the basis of the groundbreaking protein structure prediction algorithm AlphaFold2 (23).

## METHODS

### Embeddings generation

We generated the embeddings of the GPCR sequences by using a pre-trained encoder from the Evolutionary Scale Model (ESM; https://github.com/facebookresearch/esm). We computed embeddings from sequences in the Fasta format by using the *extract.py* function of the ESM library and by specifying the ESM1b model (*esm1b_t33_650M_UR50S*) with embeddings for individual amino acids as well as averaged over the full sequence using the option *‘–include mean per_tok’*.

We generated embeddings for each individual layer separately, by specifying their corresponding number in the ‘*–repr-layers*’ option. We only retained the average embedding representation for the next analysis.

### Data sets

We obtained experimental binding data from two distinct sources: the TGF assay (13), which captures the relative activities of binding of 148 GPCRs with 11 chimeric G proteins, and the EMTA biosensor (GEMTA) assay (16), which profiles the binding activities of 97 GPCRs with 12 G proteins and 3 β-arrestins/GRKs binders. We also used the Unified Coupling Map (UCM) study derived from an integrated analysis of the aforementioned assays (24), entailing binding relative activities for a total of 164 GPCRs for 14 G proteins. For the TGF assay, we considered a receptor coupled to a G protein if the logarithm (base 10) of the relative intrinsic activity (logRAi) was greater than -1, and non-coupled otherwise. Similarly, for the GEMTA assay we considered a receptor coupled to a G protein (or β-arrestins/GRK) if the double normalized Emax was greater than 0, and non-coupled otherwise. For the UCM study, we considered a receptor coupled to a G protein if the binding relative activity was greater than 0, and non-coupled otherwise.

### Model training

We trained multiple models by using embeddings obtained from the pre-trained ESM1b as features. For each of the three studies described in the previous section, we generated training matrices by taking for each receptor the mean representation of each embedding layer, which are 1280 long vectors. This yielded 1280 x *n* matrices for each training set, where *n* is the number of GPCRs in each binding set, which were subjected to Principal Component Analysis (PCA) to project them to a lower dimensional space, constituted by the number of components describing 95% of the total variance, using the function *decomposition.PCA* from Scikit-learn. Next, for each G protein/β-arrestin transducer family, we created three training matrices, each containing decomposed PCA values of the receptors in the three studies and their coupling information as classification label (see the previous section). We implemented the machine learning models using logistic regression or support vector classifier algorithms available from Scikit-learn (https://scikit-learn.org/). We performed a grid search using stratified 5-fold cross validation (CV) to select the best hyper-parameters of the algorithms. We repeated the process 10 times to ensure minimum variance. In details, we used the following hyperparameter space for logistic regression: penalties {‘l1’, ‘l2’}; solvers)‘newton-cg’, ‘lbfgs’, ‘liblinear’, ‘sag’, ‘saga’}; inverse of regularization strength(C) [0.001, 100]; maximum number of iterations (4000) and class weights (balanced). For Support Vector machines we searched for the following hyperparameter space: kernels {‘linear’, ‘poly’(three degrees), ‘rbf’, ‘sigmoid’}; kernel coefficient i.e. gamma (scale), and class weights (balanced); and the inverse of the regularization strength(C) [0.1, 100]. For each of the 17 G protein/ β-arrestin transducer families, we generated two models per embedded layer. Thus, we obtained 68 models per transducer family. We then ranked the models based on their AUC (Area Under the Curve) scores obtained during the cross-validation process. To ensure minimal imbalance, we eliminated the models with the absolute difference of Recall (REC) and Specificity (SPE) greater than 0.15 during the cross-validation process. The top 5 filtered models ranked according to AUC score were finally employed for testing on a held-out study.

To assess over-fitting, we performed a randomization test (25) by randomly shuffling the original labels of the training matrix, while preserving the ratio of the number of positive (coupled) and negative (non-coupled) GPCRs.

### Model testing

We downloaded all the known GPCR/G protein couplings provided in the GtoPDB database (http://www.guidetopharmacology.org/ (17)). A total of 117, 160, and 94 GPCR/G protein couplings were present in the GtoPDB but absent in the TGF assay (used as training set also for our original PRECOG model), the GEMTA assay, and the UCM studies, respectively. Since GtoPDB lacks a true negative set, we used Recall (REC) to compare the performance of PRECOGx with PRECOG (18). Note that we considered all human GPCRs from all the classes with the exception of Olfactory receptors, both during the training and testing stages of PRECOGx. By design, the original PRECOG and the corresponding model trained on the GEMTA assay using hand-crafted features (see below) only considered class A GPCRs. For testing β-arrestin models, we considered 57 Class A GPCR - β-arrestin 1/2 interactions obtained from STRING (combined score > 600) (26), HIPPIE (27), and IMEx (28) databases. We finally selected the 17 best performing models for each G protein or β-arrestin based on the highest Recall during testing.

As an additional test, we compared coupling probabilities with reported relative activities log(Emax/EC50) of four GPCRs (i.e. *ADRB2, NTSR1, LPAR6, HTR7*) obtained through the TRUPATH platform (15) as well as the TGFα Shedding Assay. We binarized the experimental activities, considered as ground truths, as well as coupling probabilities and we computed ACC, REC, PRE and AUC metric performances.

### PRECOG-GEMTA

As a second baseline, we trained a coupling classifier using information from the GEMTA assay by extracting the sequence-based features for G protein/β-arrestin selectivity, as described in our previous studies (18). The GEMTA assay measured binding activities of 85 Class A, 15 Class B, and 5 Class C GPCRs with 11 G proteins and 2 β-arrestins (in presence/absence of GRK2). We considered double normalized maximum value of ligand-induced response (Emax). Due to the lack of enough data for other classes, we considered only the 85 Class A GPCRs from the GEMTA assay study to develop the predictor. Briefly, we created a multiple sequence alignment, using *hmmalign* from the HMMER3 package (29), and the *7tm_1* Hidden Markov Model (HMM) from PFAM (30), of the class A GPCRs from the GEMTA assay study and subdivided the alignment based on their coupling preference (double normalized Emax) to a given interacting group (G proteins/β-arrestins) (see section Data sets). Next, we created the HMM profile of the sub-alignments using the *hmmbuild* tool from the HMMER3 package (29). To generate the training matrix, we considered the positions within the HMM profiles that showed statistically significant (p-value < = 0.05) differences in the amino acid distributions of coupled vs. non-coupled profiles. We implemented the logistic regression algorithm using the machine learning workflow as described above (see section Model training) and calculated the metrics of the best-performing model (Supplementary Table S1), which was used for prediction purposes in the current study.

### Attention head importance

By inspecting the weights of the trained classifiers, we extracted the most important attention head, for the best performing layer of each transducer family.

Let us observe that embeddings obtained through the ESM model are high-dimensional tensors $x\; \in \;{\mathbb R}^{1280}$ obtained by concatenating 64 dimensional tensors across all of the 20 attention heads of the model.

In order to compute head importance we leverage the linear structure of the classification pipeline. In more depth, our pipeline maps an input embedding $x$ to a lower dimensional representation *z*:

(1) $$\begin{equation*}z\; = \left[ {{z_1}, \ldots ,{z_K}} \right]\; \in {{\mathbb R}^K}\;\;{z_i} = \;\mathop \sum \nolimits_{j\; = \;1}^{1280} w_{i,j}^{PCA}{x_j}\end{equation*}$$

through PCA, where $K$ is the optimal number of components chosen through cross-validation as described in the model training section. Each classifier than computes a score *S(x)*:

(2) $$\begin{equation*}S\;\left( x \right) = \;\mathop \sum \nolimits_{k\; = \;1}^K w_k^{cl}{z_k}\end{equation*}$$

which is then transformed into a class probability. In order to obtain head importance, we mapped back to $x$ importance weights from the final classifier through PCA. We defined the importance of the $k$-th weight (associated with the $k$-th PCA component) of the classifier as:

(3) $$\begin{equation*}{\rm{\;}}\bar w_k^{cl} = \;\frac{{\left| {w_k^{cl}} \right|}}{{\mathop \sum \nolimits_{k\; = \;1}^K \left| {w_k^{cl}} \right|}}\end{equation*}$$

Moreover, we defined the importance of the original $i$-th element of $x$ in the $k$-th component of PCA as:

(4) $$\begin{equation*}{\rm{\;}}\bar w_{i,k}^{PCA} = \;\frac{{\left| {w_{i,k}^{PCA}} \right|}}{{\mathop \sum \nolimits_{j\; = \;1}^{1280} \left| {w_{i,j}^{PCA}} \right|}}\end{equation*}$$

The quantities defined allow us to compute a reweighted principal component matrix as follows

(5) $$\begin{equation*}\left( {\begin{array}{@{}*{3}{c}@{}} {\bar w_1^{cl}\bar w_{1,1}^{PCA}}& \cdots &{\bar w_1^{cl}\bar w_{1280,1}^{PCA}}\\ \vdots & \ddots & \vdots \\ {\bar w_k^{cl}\bar w_{1,k}^{PCA}}& \cdots &{\bar w_k^{cl}\bar w_{1280,k}^{PCA}} \end{array}} \right)\end{equation*}$$

Recalling that each head outputs a 64 dimensional representation, the $h$-head's importance is then obtained as:

(6) $$\begin{equation*}{\rm{\;}}{I_h} = \;\mathop \sum \nolimits_{k\; = \;1}^K \mathop \sum \nolimits_{j\; = \;l*64 + 1}^{\left( {l + 1} \right)*64} \bar w_k^{cl}\bar w_{j,k}^{PCA}\end{equation*}$$

By varying $h$ from 1 to 20 we obtained vector $I\; = \;[ {{I_1}, \ldots ,{I_{20}}} ]$ and identify the most important head by selecting the head with maximum importance value. If the best performing model was obtained using the support vector classifier (with a non-linear kernel), we resorted to the logistic regression model (trained on the same embedding layer and assay study) to compute the most important head.

### Unsupervised learning of the GPCRome embedded space

We generated embeddings for the human GPCRome, comprising a total of 377 receptors (287 Class A, 15 Class B1, 17 Class B2, 17 class C, 11 class F, 25 Taste receptors and 5 in other classes) shorter than 1024 amino acids in length due ESM model length constraints. We performed Principal Component Analysis (PCA) on each embedding layer using the *PCA* function from *decomposition.PCA* method of the Scikit-learn package (https://scikit-learn.org/). Each human GPCR sequence was annotated with the available functional labels, i.e. GtoPdb Class membership or Transduction Mechanism, couplings from the TGFα shedding or GEMTA and STRING interactions (for β-arrestins).

We performed K-means clustering of the study projected along the first two components of the PCA using the function *cluster.KMeans* from Scikit-learn. The number of clusters was set as the number of variables possible for the given functional label (i.e. GtoPdb GPCR Class, Transduction mechanisms or Coupling specificities from either TGF or GEMTA assays). In the case of GtoPdb class information, the number of clusters was set to 5 (possible variables: Class A, Class B, Class C, Frizzled, and Taste). For the remaining functional labels about coupling information, the number of clusters was set to 2 (possible variables: coupled or non-coupled to a G protein/β-arrestin). We then calculated the Normalized Mutual Information (NMI) score of the resulting clusters using *metrics.cluster* from Scikit-learn for all the 33 layers. We chose the best layer for a given functional label as the one with the highest NMI score.

### Contact analysis

To interpret the determinants of G protein binding specificity, we first calculated predicted intra-molecular contacts for each receptor sequence using the logistic regression algorithm trained over the ESM’s attention maps, (using the function *predict_contacts* in the ESM library) (31) and retaining predicted contact with a probability greater than 0.5. We referenced sequence residue positions to the GPCRdb generic residue numbers (32). Next, contact maps were grouped based on G protein binding specificity (either TGF, GEMTA or UCM) and differential contact maps were derived by calculating the log-odds ratio from the following contingency Table 1:

**Table 1.** Contingency table for calculating log-odds ratio

Contact pair/G protein	Contact	No contact
Coupled	AA	BB
Not coupled	CC	DD

using the following equation (1):

(7) $$\begin{equation*}{\rm{log - odds ratio}}\,{\rm{ = }}\,{\rm{log}}\,\left( {\frac{{AA}}{{DD}} \times \frac{{CC}}{{BB}}} \right)\end{equation*}$$

AA and BB terms represent a number of coupled GPCRs to a specific G protein depending on the assay that have or do not have a specific contact pair, respectively. CC and DD terms represent the number of non-coupled GPCRs for a specific G protein depending on the assay, that has or does not have a specific contact pair respectively. Contacts contributed from the loops, N-termini and C-termini of the GPCR where aggregated. We calculated the enrichment in a specific transducer family with respect to non-coupled receptors for a consensus list of 223 unique pairs, corresponding to 181 unique GPCRdb positions for the UCM study (220 and 184 unique pairs and positions for the GEMTA assay or 231 and 186 unique pairs and positions for the TGF assay).

We computed log-odds ratio using the Table2×2 function from StatsModels (https://www.statsmodels.org/). Resulting log-odds ratios were normalized using the *MaxAbsScaler* from scikit-learn.

Contacts with a positive log-odds ratio (enriched) are seen more frequently in receptors coupled to a specific G protein, while contacts with a negative log-odds ratio (depleted) are seen less frequently in receptors coupled to a specific G protein.

### GPCR-Gα complexes prediction via AlphaFold-Multimer

A total of 2141 GPCR-Gα pairs, reported to bind in either GtoPdb or the TGF assay or the GEMTA assay, were considered, respectively corresponding to 265 and 14 human GPCRs and Gα proteins (the three members of the *GNAT* family are not considered). We generated through Alphafold-Multimer v2.1.1 (33) the 3D structural models for each of these experimental GPCR-Gα complexes lacking a known 3D structure in the PDB. The databases required to run AlphaFold-Multimer were downloaded on 16 November 2021. Among the 5 models generated for each GPCR-Gα pair, only the one with the highest confidence was considered for further analysis.

### ClinVar mutations analysis

We used PRECOGx to predict the functional consequences of 2140 missense variants (212 GPCRs) from ClinVar (34). For each variant we compared predicted couplings with the ones calculated for the wild-type receptor sequence. Variants or wild-types with predicted probability higher than 0.5 were considered coupled and those with lower probability as uncoupled to specific G protein.

### Healthy Tissue (GTEx) alternative splicing isoforms

We used PRECOGx to predict the impact of alternative splicing on GPCR signalling. We considered 1141 protein-coding, alternatively spliced mRNA transcripts from 364 unique genes from GTEx (35). We used the best performing model (PRECOGx) to profile coupling specificities for both canonical and spliced variants. Spliceforms with predicted probability greater than 0.5 were considered coupled and non-coupled otherwise. We annotated spliceforms with their highest expression across the tissue. Different conditions were tested by imposing cutoffs for isoform length (i.e. retaining 25%,50% 75% or all of their 7TM segments) or for expression (TPM > = 1.0).

### Pipeline

**Figure 1.**
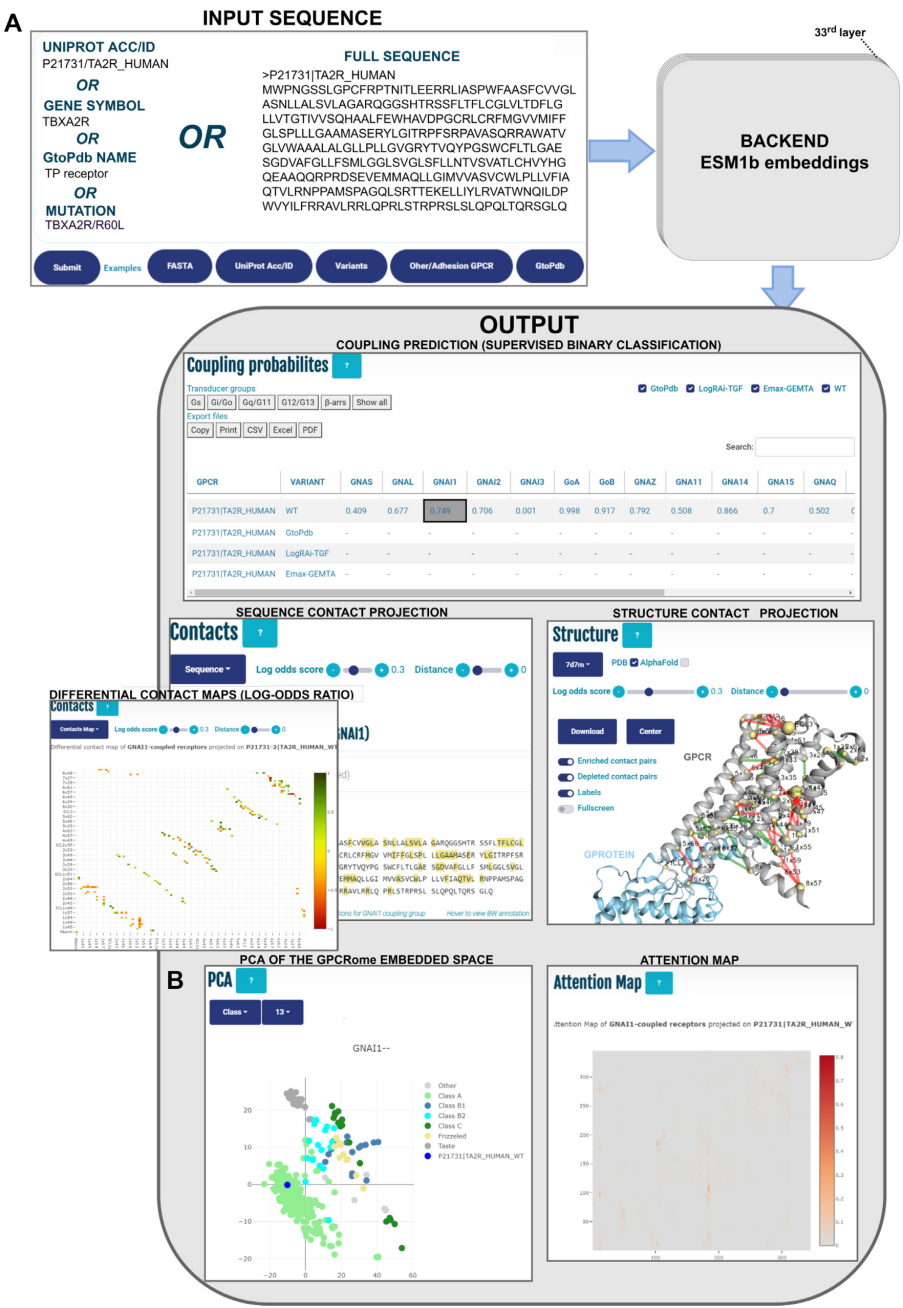
(**A**) Workflow of PRECOGx web application. The input can be provided in different formats, including: protein identifiers (UniProt identifiers, accessions, gene symbols or GtoPdb official nomenclatures), mutations in the format protein identifier/aa substitution (e.g. MC1R/D294H) or FASTA sequences. In either cases, the corresponding sequences are used to create ESM embeddings which are in turn employed in different backend processes to generate PRECOGx output. The latter is presented in a multi-panel view including: summary table of the predicted and known couplings, predicted differential contacts mapped in 1D (Sequence panel), 2D (Contact panel) or 3D (Structure panel), attention maps and (**B**) K-means clustering based on GPCR class of the entire GPCRome projected along the first two components of the PCA. The number of clusters was set to 6 (possible variables: Class A, Class B1, Class B2, Class C, Frizzled, and Taste).

Given user input data, i.e. receptor WT or mutant sequences, the web server backend generates the ESM embedding features (see Figure 1). The average embeddings are extracted and the ones corresponding to the best performing layer for the classification of a given coupling are used as features in the corresponding model for coupling classifications. The embedding layers are also used to project the input sequence in the PCA embedded space previously generated for the human GPCRome.

To detect the closest homolog for structural visualization purposes, every input sequence is aligned through PSI-BLAST (36) either to 3D structures of GPCRs G protein/β-arrestin complexes from the PDB or to AlphaFold-Multimer predicted complexes. Identified matches are returned for visualization and sorted based on percentage of identity. Sequence and structure residue positions were referenced to the GPCRdb generic residue numbers from GPCRdb (32).

We developed PRECOGx using Apache2 (https://httpd.apache.org/) using the Python programming language, both for the web frontend, which is based on Flask (http://flask.pocoo.org/) and for the internal pipeline to handle back-end processes. We additionally used the following Python and JavaScript libraries at both back- and front-ends: NGL Viewer (v1.3.1), jQuery (v3.5.1), neXtProt (v0.2.17), Bootstrap (v5.13), Scikit-learn (v1.0.2), DataTables (v1.11.3), Plotly (v2.6.3).

## RESULTS

### Using the webserver

The input can be one or more protein identifiers (UniProt identifiers, accessions, gene symbols or GtoPdb official nomenclatures), mutations in the format protein identifier/aa substitution (e.g. MC1R/D294H) or FASTA sequences (see Figure 1A). Examples of the different inputs accepted are available through dedicated buttons besides the ‘Submit’ one. The mutation format is particularly suited for predicting the functional consequence of missense mutations. For larger variants, e.g. alternative splicing variants, the user is recommended to directly input the corresponding FASTA sequence (see section ‘Predicting the functional consequences of GPCRs variants.’ below).

On the results page, a tutorial is available on the top. Predictions for each individual G protein/β-arrestin are tabulated in the upper panel. Each row lists either predicted coupling probabilities or experimental binding data, whenever available, for each given input. In the centre-left of Figure 1A, predicted intra-molecular contacts are displayed at the primary sequence level. Alternatively, transducer family-specific predicted contacts are shown via a toggle button on a heatmap, where cells are colored according to enrichment (green = enriched; red = depleted). In the centre-right, the predicted intra-molecular contacts are highlighted on a 3D structure with edges colored according to coupling specificity (green = enriched; red = depleted) and contacting residues shown as spheres whose radius is proportional to their contact network degree, by default the one best-matching (via BLAST) the input. The visualized structures can be optionally changed and, alternatively to experimental PDB structures, 3D models predicted via AlphaFold-Multimer can also be visualized. On the bottom-left, a PCA plot of the GPCRome sequence space is used to project and track the location of the input sequence (Figure 1B). This new feature performs PCA and k-means clustering on ESM embeddings of the non-olfactory human GPCRome to generate a low dimensional space where any input sequence can be projected and analysed. For instance, it is possible to input the wild type sequence of a GPCR (e.g. human *TBXA2R*, blue dot in Figure 1B) and perform the PCA projections on a specific embeddings layer to uncover functional patterns. To ease pattern detection, points corresponding to reference human GPCRome receptors can be colored based on functional information via a drop-down menu which allows to specify either GtoPdb class or transducer coupling mechanisms from either GtoPdb, TGF or GEMTA studies. For instance, the 13^th^ layer is the one leading to the GPCRome clustering that best agrees with the GPCR Class annotation according to the NMI score metric (Figure 1B; see Methods). PCA bi-dimensional representation of the embedded space can also be used to visualise the trajectories of natural or artificial variants with respect to the reference GPCRome sequence space (see below). In the bottom-right, attention maps from the most informative attention head of a given layer can be visualised to explore residue-residue dependencies associated to a given coupling.

The information displayed in the sequence, 2D and 3D contacts visualizations as well as in the attention map panels is automatically updated by clicking on individual cells of the prediction table corresponding to a specific receptor-transducer pair (see below for more use examples of these panels).

### Protein language models are predictive of GPCRs signalling mechanisms: beyond class A and G proteins

**Figure 2.**
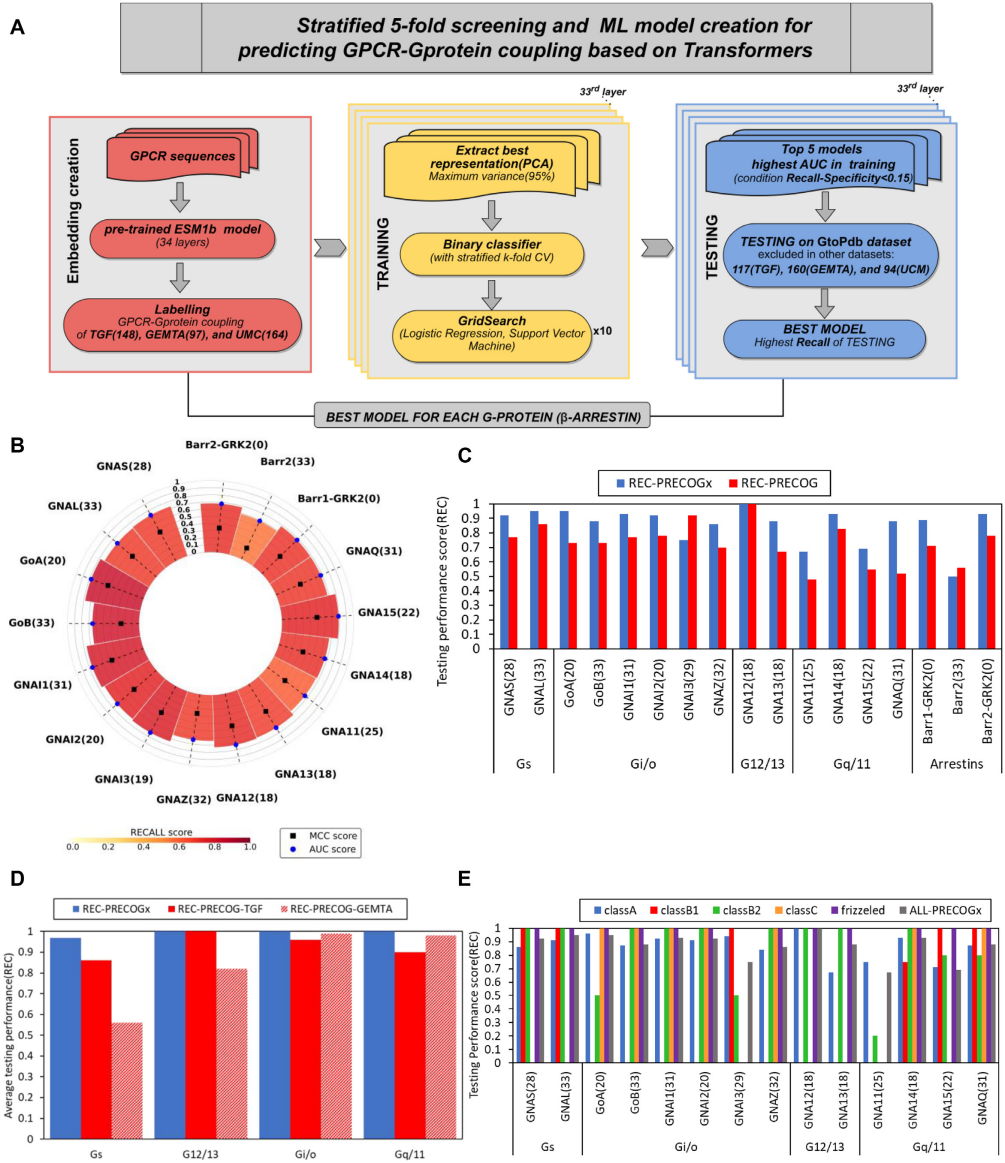
PRECOGx ML model creation and screening. (**A**) Workflow of ML model creation for predicting GPCR-Gprotein (or β-arrestin) coupling; (**B**) 5-fold CV parameters (AUC,REC,MCC) of best performing models for specific Gprotein (or β-arrestin); (**C**) Comparison of testing performance (REC) of PRECOGx with PRECOG for each transducer. Depending on whether the TGF assay or the GEMTA assay returned the best performing model in PRECOGX for a given interaction, we compared with either the original PRECOG (trained on TGF) or PRECOG-GEMTA; (**D**) Comparison of testing performance (REC) of PRECOGx and previously created PRECOG across each Gprotein family; (**E**) Comparison of testing performance (REC) of PRECOGx across different GPCR classes.

We trained and tested multiple machine learning models by considering different combinations of embedding layers, algorithms and training sets. The best performing model for every interacting group, for a total of 17 G proteins/β-arrestins partners, was selected based on the AUC during the training and REC during testing phase (Figure 2A, B; Supplementary Table S2; Methods). Models showed overall good stability, with low standard deviations of the performance metrics, and minimal overfit, with training on randomly shuffled labels always performing worse (Supplementary Figure S1A). With respect to coupling specificity, we found that seven best performing models were obtained by training on the Shedding assay and six by the GEMTA assay. The latter included three G proteins (i.e. GoB, *GNAI2* and *GNA11*), and β-arrestins, whose binding data were not included in the TGF assay (Supplementary Table S2).

The best-performing models (collectively called PRECOGx) were tested on an independent test set comprising GPCRs that were absent in the training set but have known G protein coupling information reported in the literature (i.e. GtoPDB (17)). To test the predictions for β-arrestins, we considered high-confidence interactions from functional interaction databases (see Methods). We compared the performance of PRECOGx with the previous PRECOG approach trained on TGF as well as on the GEMTA studies (respectively termed PRECOG and PRECOG-GEMTA;see Methods). With the only exception of *GNAI3* and β-arrestin2, PRECOGx outperformed PRECOG-based models (Figure 2C). This trend is evident also when aggregating the recall metric family-wise, particularly for G_s_ (Figure 2D).

We also trained the models based on the Unified Coupling Map study generated by intersecting the TGF and the GEMTA studies (37). The model trained on the UCM study performed overall worse than the one trained on the individual sets (Supplementary Figure S1B; Supplementary Table S3). Notably, while the original PRECOG was limited by design only to class A receptors, PRECOGx can be used to predict coupling specificities of any receptor regardless of its class. In particular, PRECOGx is able to recapitulate well known G_s_ preferences for several class B receptors, G_i/o_ for class C and G_12/13_ and G_q/11_ for Frizzled receptors (Figure 2E; Supplementary Table S4). To further validate the model, we have also compared PRECOGx predictions with reported couplings of four receptors from the TRUPATH platform (15) (Supplementary Table S5). A total of 112 non-olfactory GPCRs, corresponding to 28% (112 out of 393) of the human GPCRome, have reported coupling neither in GtoPDB nor in quantitative binding studies. We now provide a comprehensive repertoire of predicted couplings for the entire non-olfactory, human GPCRome(Supplementary Table S6). For example, the model is able to correctly predict *TASR1* and *TASR2* coupling preference for *GNAI1* and GoA. These receptors are the members of T1R family of taste receptors which are involved in the detection of sweet-tasting compounds and have been shown to preferentially couple aforementioned G proteins (38). We also successfully predicted coupling preference for *GNAI2* of *TAS2R16*, a taste receptor with a role in bitter-tasting shown to signal mainly through with *GNAI2* in a Ric-8A mediated fashion (39).

### Predicted intra-molecular contacts inform about transducer family specific signatures

**Figure 3.**
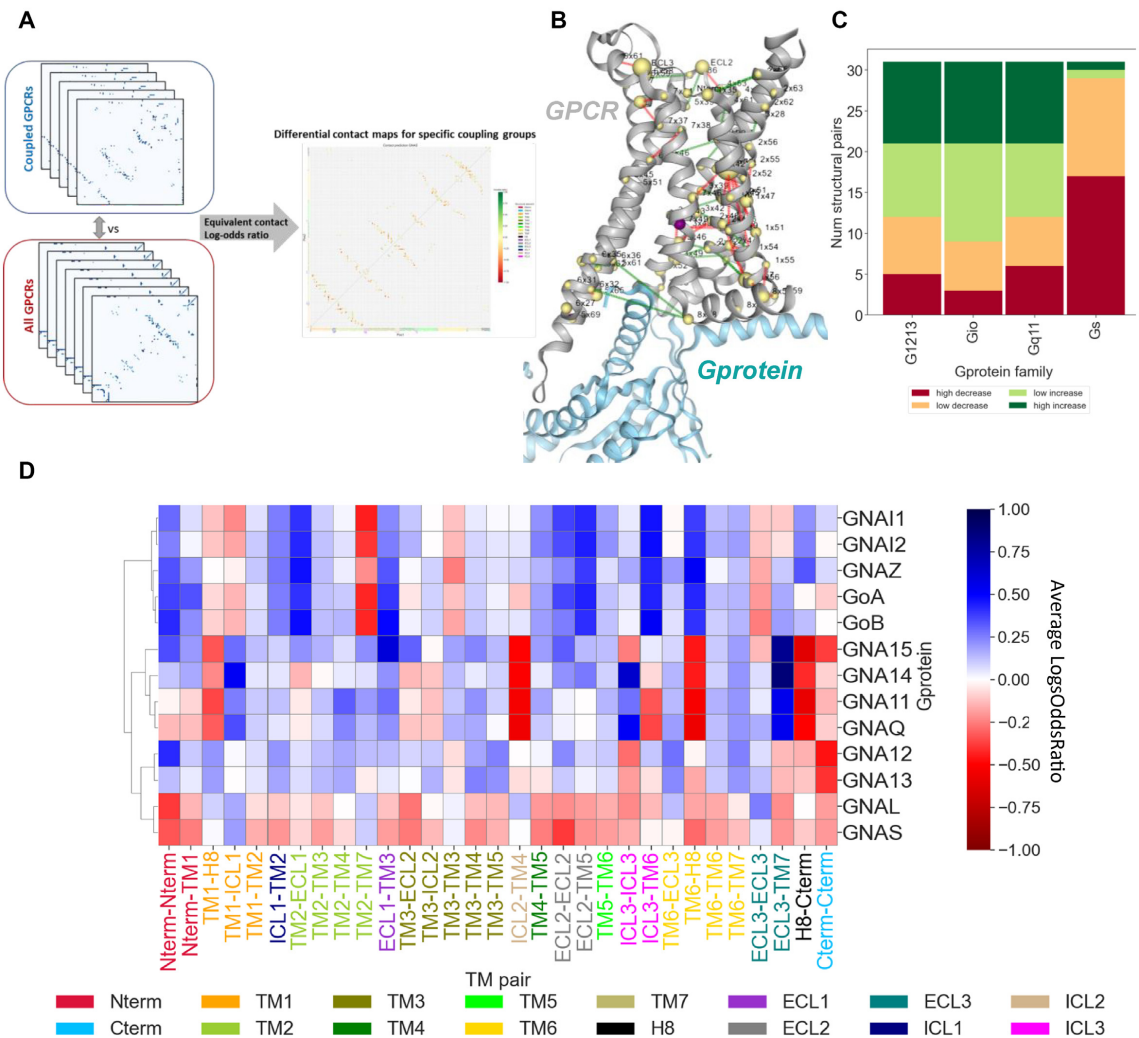
Differential contact analysis: (**A**) Calculation of predicted contacts of each GPCR using the function predict_contacts in the ESM library and grouping GPCR based on G protein binding specificity to compute differential contact maps (log-odds ratio); (**B**) Projection of transducer family-specific predicted contacts on a structure in PRECOGx application (PDB 7JOZ); (**C**) Number of aggregated enriched(increase) or depleted(decreased) secondary structure element pairs for each Gprotein transducer family; (**D**) Contact enrichment signature of each Gprotein transducer family on the basis of secondary structure element.

We employed the ESM contact prediction model to predict 3D intra-molecular contacts for each GPCR sequence and used transduction information to compute differential, intra-molecular contact maps (see Methods). This yielded a bi-dimensional contact enrichment map which allows identifying contacts between secondary structure elements differing among transducer families (Figure 3A). Differential contact maps can be visualized on a 3D structure to highlight the intramolecular interactions most associated with a give coupling class (Figure 3B). Bi-dimensional contact maps can be linearized and aggregated on the basis of secondary structure elements to obtain a contact enrichment signature for each G protein transducer family (Figure 3D). We used these signatures to cluster the transducer families on the basis of their similarity which recapitulated the family membership and moreover highlighted the structural features responsible for a specific coupling, either at the individual gene or family level (Figure 3D). Every transducer family retains a highly specific intra-molecular contact signature. For instance G_s_ members are depleted in contacts at multiple regions, including the selectivity filter (40) formed by TM5 and TM6 regions flanking ICL3 (TM5-TM6 and ICL3-TM6), which is instead enriched in G_i/o_ members (Figure 3D). Overall, G_s_ receptors are characterised by a larger fraction of depleted intra-molecular contacts (Figure 3C), supporting evidences that G_s_ binding is associated with lower structural constraints and higher structural plasticity to accommodate the bulkier G_s_ C-terminal tail at the receptors binding crevice (40).

### Predicting the functional consequences of GPCRs variants

**Figure 4.**
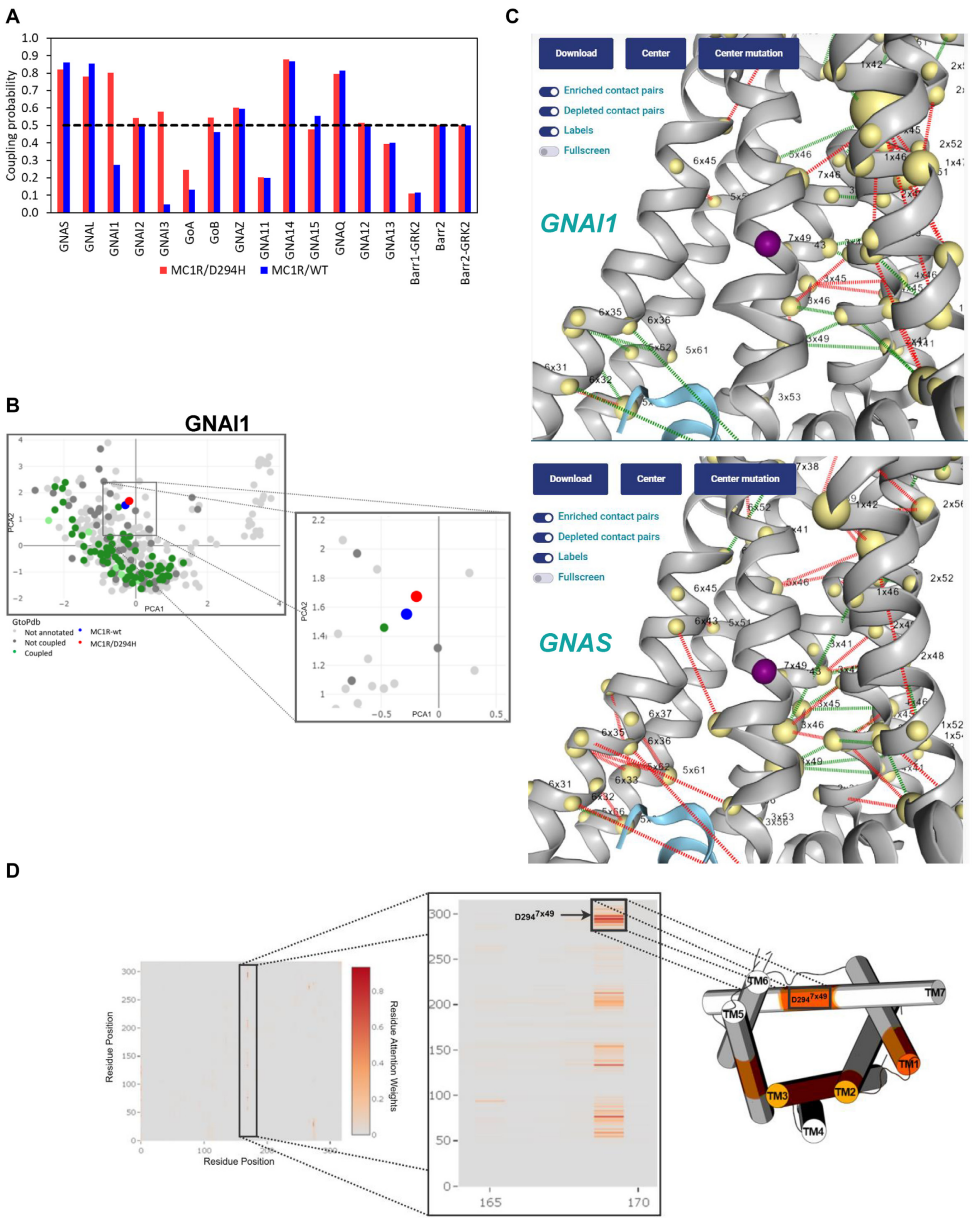
(**A**) Prediction of a missense mutation MC1R/D294H in PRECOGx(upper left); (**B**) PCA projection of the mutation in GPCRome PCA sequence space(down left); (**C**) Projection of the mutation on a 3D structure along with predicted contacts for *GNAI1*(upper right) or *GNAS* (down right) G proteins; (**D**)left-panel: attention map of the most important attention head of the best layer for *GNAI1* binding prediction; mid-panel: zoom caption of the attention signatures involving the mutated residue (i.e. D294H); right-panel: 3D cylindrical cartoon model (PDB: 7f58) representation of the residue regions involved in attention networks with the D294H site.

We show applications of PRECOGx to interpret the functional consequences of GPCRs either disease mutations or alternative splicing variants. We predicted the functional consequences of 2470 missense variants (for 214 unique GPCRs) from ClinVar with PRECOGx (Supplementary Table S7). We have also predicted the effects of interface mutations known to affect the interaction of G proteins with GPCRs (Supplementary Table S8). Whenever a mutation is inputted, PRECOGx calculates the coupling probabilities for both the mutant and wild type forms (Figure 4). By comparing predicted couplings for the variants with the corresponding wild-types it is possible to identify the mutations leading to a switch in coupling, i.e. either gain (i.e. mutant coupled vs. WT uncoupled) or loss (mutant uncoupled vs. WT coupled) (Figure 4A; Supplementary Table S7;Methods). For example, the variant MC1R p.D294H^7x49^ (dbSNP id: *rs1805009*; superscript refers to GPCRdb generic residue numbers) is classified as a risk factor for melanoma and is reported to lose the capability to stimulate cAMP levels (41). PRECOGx predicted that this mutation enhances the coupling towards several G_i/o_ family members, suggesting that the reduced cAMP levels might follow an increased inhibition of Adenylate Cyclase via G_i/o_ coupling (Figure 4A). Projections of the embeddings of the mutated sequence on the GPCRome embedded space allows the user to visualize the trajectory of the mutant with respect to the WT form (Figure 4B). Visualization of the mutation site in the structure panel of web interface allows the user to inspect the mutation site in the context of the coupling specific contact network which differentiates *GNAI1* from *GNAS* (Figure 4C). Moreover, visualization of the attention map derived from the most important attention head of the best performing layer during classification, allowed us to interpret the effect of the D294^7x49^ mutation, which participate to a characteristic attention signature impinging on residue 170 (Figure 4D).

**Figure 5.**
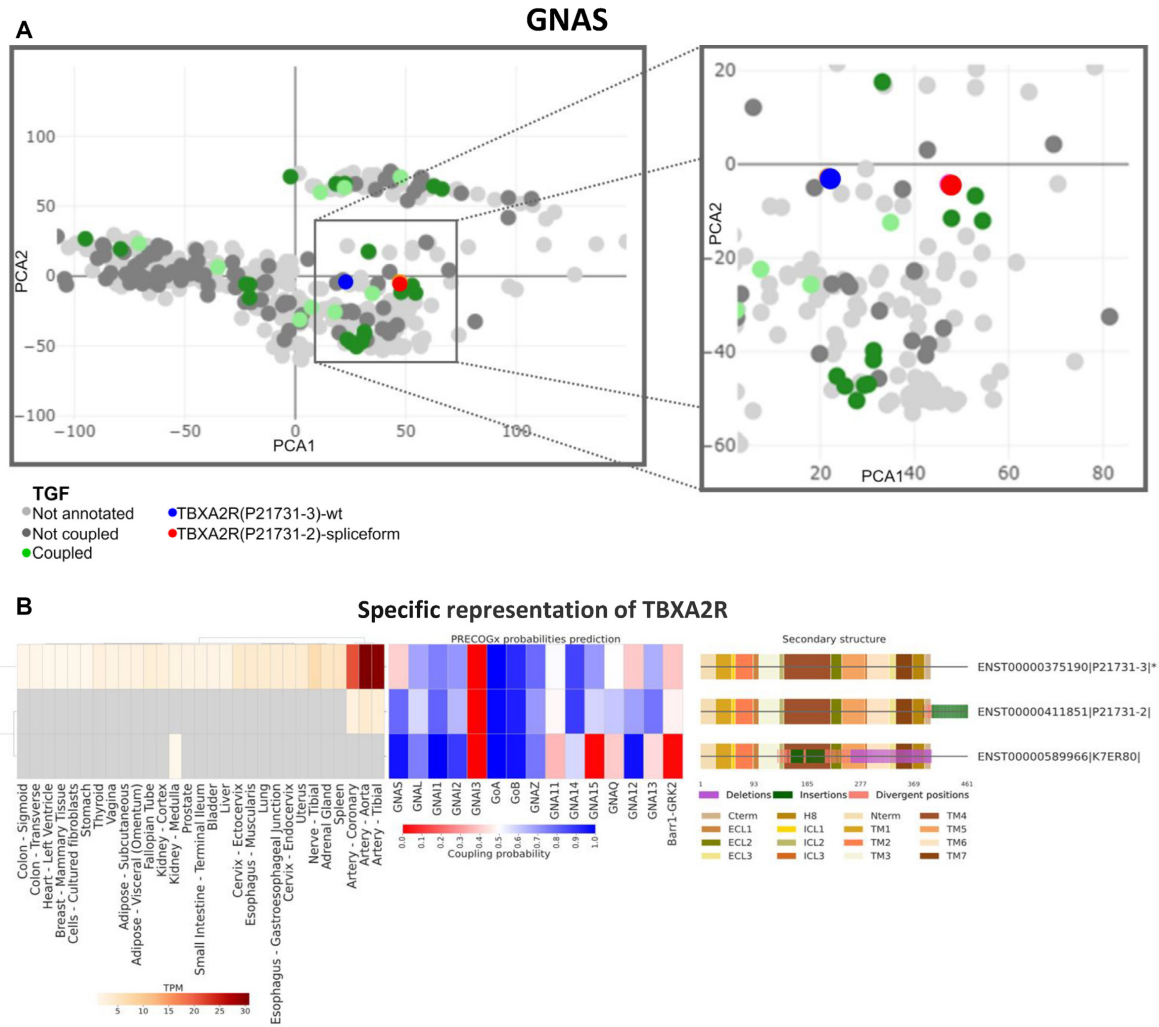
Predicting GPCRs alternative splicing signalling consequences: (**A**) PCA projection of TBXA2R canonical sequence and its spliceform in GPCRome embedded space; (**B**) Visualization of tissue expression in GTEx, PRECOGx predictions and structural differences between canonical sequence(*) and spliceform sequences for *TBXA2R*.

As an additional use case, we used PRECOGx to predict the impact of alternative splicing on GPCR signalling. We considered a total of 1141 protein-coding, alternatively spliced mRNA transcripts from 364 unique genes from GTEx (35) and we used the classifier to profile both canonical and alternatively spliced variants for binding probabilities. A total of 265 alternative splicing transcripts were predicted to change coupling classification (either gain or loss of binding with respect to the canonical sequence) for at least one binding partner (either G proteins or β-arrestins), out of which 105 were expressed in at least one tissue with an abundance equal or greater than 1TPM (Supplementary Table S9). Among our hits, we found several spliceforms previously reported to alter intracellular signalling (Supplementary Table S9) (42). For example, the *TBXA2R* alternative spliceform 2 is predicted to gain *GNAS* coupling with respect to the canonical one (Figure 5A, B). The PCA panel illustrates this functional effect by showing the *TBXA2R* spliceform 2 (red dot) approaching a cluster of *GNAS* coupled receptor (green dots) with respect to WT *TBXA2R* (blue dot; Figure 5A). While variation at the C-terminal is most often predicted to alter intracellular signalling, we predicted that also certain N-terminal variants might perturb intracellular signalling via allosteric mechanisms (Supplementary Figure S2). For example a N-terminal splice variant of *GHRHR*, which has been shown to alter the signalling properties (i.e. G_s_ vs β-arrestins), is also predicted to mildly alter corresponding couplings (Supplementary Table S9) (43).

## DISCUSSION

We present a new method, called PRECOGx, to predict GPCRs coupling specificities which represents an improvement over its predecessor (PRECOG (18)). Our previous approach was trained on hand-crafted features comprising sequence-based descriptors, either from the 7TM bundle or the intra-cellular loops, which were found to be statistically associated with a certain coupling. This set of features was discrete, encompassing a few regions of the 7TM architecture, and was highly tailored to the experimental binding study that we used to train the model (i.e. 144 Class A GPCRs from the TGF assay). A key addition to this new resource is the use of protein embeddings derived from state-of-art protein language model (ESM1b) which has been pre-trained on hundreds of millions sequences. ESM embeddings encode intra-sequence amino acids contextual dependencies which have been shown to well recapitulate the structure and function of proteins (19). We therefore exploited the generalisability of this model to obtain deep, numerical representations for all human GPCRs, which allowed us to model the signalling properties of receptors from classes other than A, which were excluded from our previous analysis. The performances of PRECOGx for all the GPCR classes are even more remarkable if we consider that the training sets that we employed are generally enriched in Class A members.

The construction of our model entailed a critical and systematic assessment of the predictive power of classifier algorithms trained on distinct quantitative binding studies, such as TGFα shedding or GEMTA. While performances are overall comparable, we observe that optimal outcomes for certain interactors are study specific (e.g. *GNA15* based on GEMTA assay or *GNAS* based on Shedding; Supplementary Table S2), suggesting that certain experimental settings might be more accurate and lead to more generalizable models for specific interaction partners. It is also possible that the observed slight differences might be due to intrinsic differences of the assays and generated binding data as well as to the different specific cutoff choices employed.

One clear advantage of the previous classifier was its inherent interpretability due to hand-crafted features. On the other hand, interpretability of transformers models such as the ESM is still an open area of research (44). Here we addressed this issue by outputting for each best performing embedding layer for a given coupling partner the attention map of the head receiving higher weights in the model, which is instrumental in understanding receptor's residue contextual dependencies associated with a certain coupling. Moreover, we also computed a map of differentially predicted contacts which allows us to visualize the intramolecular contacts recurring for certain couplings. We noted that different layers, encoding different contextual properties, are associated with different couplings. Understanding the structural, dynamical and functional nature of these couplings will be a matter of future investigations.

The new method also allows to predict the effect of mutations at virtually any position within the sequence as well as it can deal with larger variation such as splicing variants. On one hand, it can complement ongoing efforts to catalogue the functional impact of the myriad of cancer somatic mutations observed in GPCRs (7,45). On the other hand, our approach can provide mechanistic interpretation to recent systematic analysis showing the widespread role of alternative splicing to modulate GPCR signalling in healthy tissues (42). We also provide novel functionalities in the webserver frontend, such as the PCA panel, which allows the user to visualize the trajectories of variants with respect to a reference low-dimensional sequence space of the human GPCRome. Future efforts will focus on using more robust pre-trained models to account for mutation effects at the interaction interfaces with both the ligands as well as the transducers as well as within the network of intra-molecular contacts governing allosteric transitions.

In summary, the novel PRECOGx functionalities will be of great help to better understand GPCR signalling mechanisms, to interpret GPCRs disease variants, as well as to assist future receptor design efforts.

## DATA AVAILABILITY

PRECOGx webserver is freely available at: https://precogx.bioinfolab.sns.it/.

The underlying code is freely available at: https://github.com/raimondilab/precogx.

## Supplementary Material

gkac426_Supplemental_Files
