# Supplementary material for: PRECOGx: exploring GPCR signaling mechanisms with deep protein representations
Source: Nucleic Acids Res. 2022 May 26;50(W1):W598–610. doi: 10.1093/nar/gkac426 (PMC9252787; doi:10.1093/nar/gkac426)
Supplement: gkac426_Supplemental_Files [file gkac426_supplemental_files.zip › Supplementary_Material.pdf]

PRECOGx: exploring GPCR signaling mechanisms with deep protein representations

(precogx.bioinfolab.sns.it)

Marin Matic[1],* Gurdeep Singh[2]* , Francesco Carli[1], Natalia De Oliveira Rosa[1], Pasquale Miglionico[1], Lorenzo Magni[1], J. Silvio Gutkind[3], Robert B. Russell[2] , Asuka Inoue[4] and Francesco Raimondi[1+]

Supplementary Material

Supplementary Figure Legends

Figure S1: A) Salberg randomization test results; B) Comparison of testing performance(REC) of meta-coupling dataset and previously created PRECOG for each Gprotein. C) Comparison of testing performance (REC) of meta-coupling dataset across different GPCR classes.

Figure S2: alternative spliceform predicted effect at different secondary structure elements.

Supplementary Tables

Table S1: Performance during the training phase (5-fold-cross validation) of the PRECOG approach on the GEMTA assay dataset

Table S2: model performances during training on individual quantitative datasets (TGFor GEMTA) datasets A1and testing on unseen GtoPdb couplings

Table S3: model performances during training on the UCM dataset and testing on unseen GtoPdb couplings

Table S4: performances of model testing for distinct GPCR classes+A1

Table S5: testing of PRECOGx on four reported couplings from TRUPATH and TGF assays

Table S6: PRECOGx prediction for GPCRs whose transduction mechanism is not reported in GtoPDB

Table S7: predicted coupling class switch for GPCR ClinVar mutations with respect to WT sequences

Table S8: predicted coupling effect for GPCR-G protein interface mutations from Gproteindb (https://gproteindb.org/)

Table S9: alternative splicing isoforms predicted to change at least one transducer family with respect to canonical isoform; Coupling probabilities are reported as delta of the alternative isoform with respect to the canonical isoform.

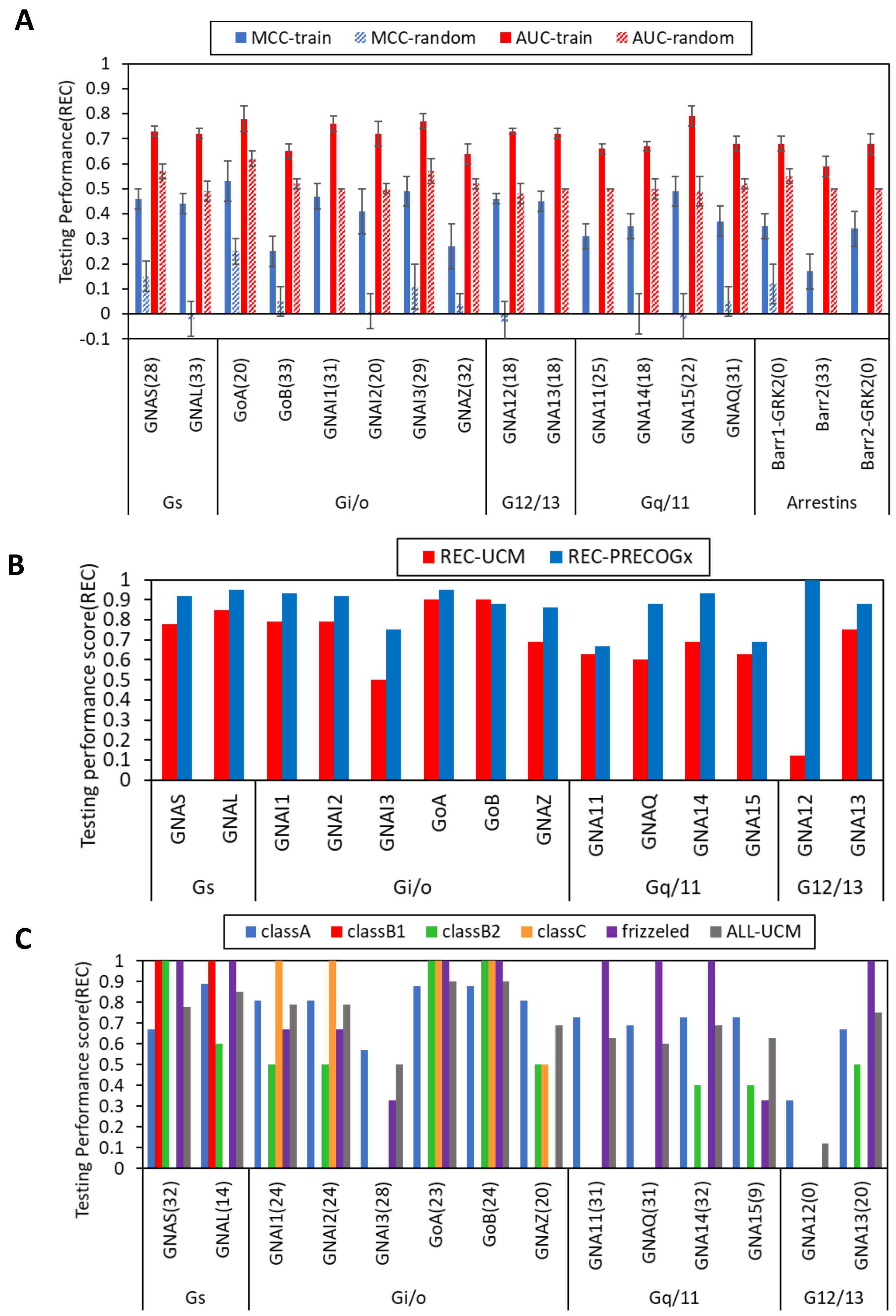
A
MCC-train
MCC-random
AUC-train
AUC-random
Testing Performance(REC)
1
0.9
0.8
0.7
0.6
0.5
0.4
0.3
0.2
0.1
0
-0.1
GNAS(28)
GNAL(33)
GoA(20)
GoB(33)
GNAI1(31)
GNAI2(20)
GNAI3(29)
GNAZ(32)
GNA12(18)
GNA13(18)
GNA11(25)
GNA14(18)
GNA15(22)
GNAQ(31)
Barr1-GRK2(0)
Barr2(33)
Barr2-GRK2(0)
Gs
Gi/o
G12/13
Gq/11
Arrestins
B
REC-UCM
REC-PRECOGx
Testing performance score(REC)
1
0.9
0.8
0.7
0.6
0.5
0.4
0.3
0.2
0.1
0
GNAS
GNAL
GNAI1
GNAI2
GNAI3
GoA
GoB
GNAZ
GNA11
GNAQ
GNA14
GNA15
GNA12
GNA13
Gs
Gi/o
Gq/11
G12/13
C
classA
classB1
classB2
classC
frizzeled
ALL-UCM
Testing Performance score(REC)
1
0.9
0.8
0.7
0.6
0.5
0.4
0.3
0.2
0.1
0
GNAS(32)
GNAL(14)
GNAI1(24)
GNAI2(24)
GNAI3(28)
GoA(23)
GoB(24)
GNAZ(20)
GNA11(31)
GNAQ(31)
GNA14(32)
GNA15(9)
GNA12(0)
GNA13(20)
Gs
Gi/o
Gq/11
G12/13

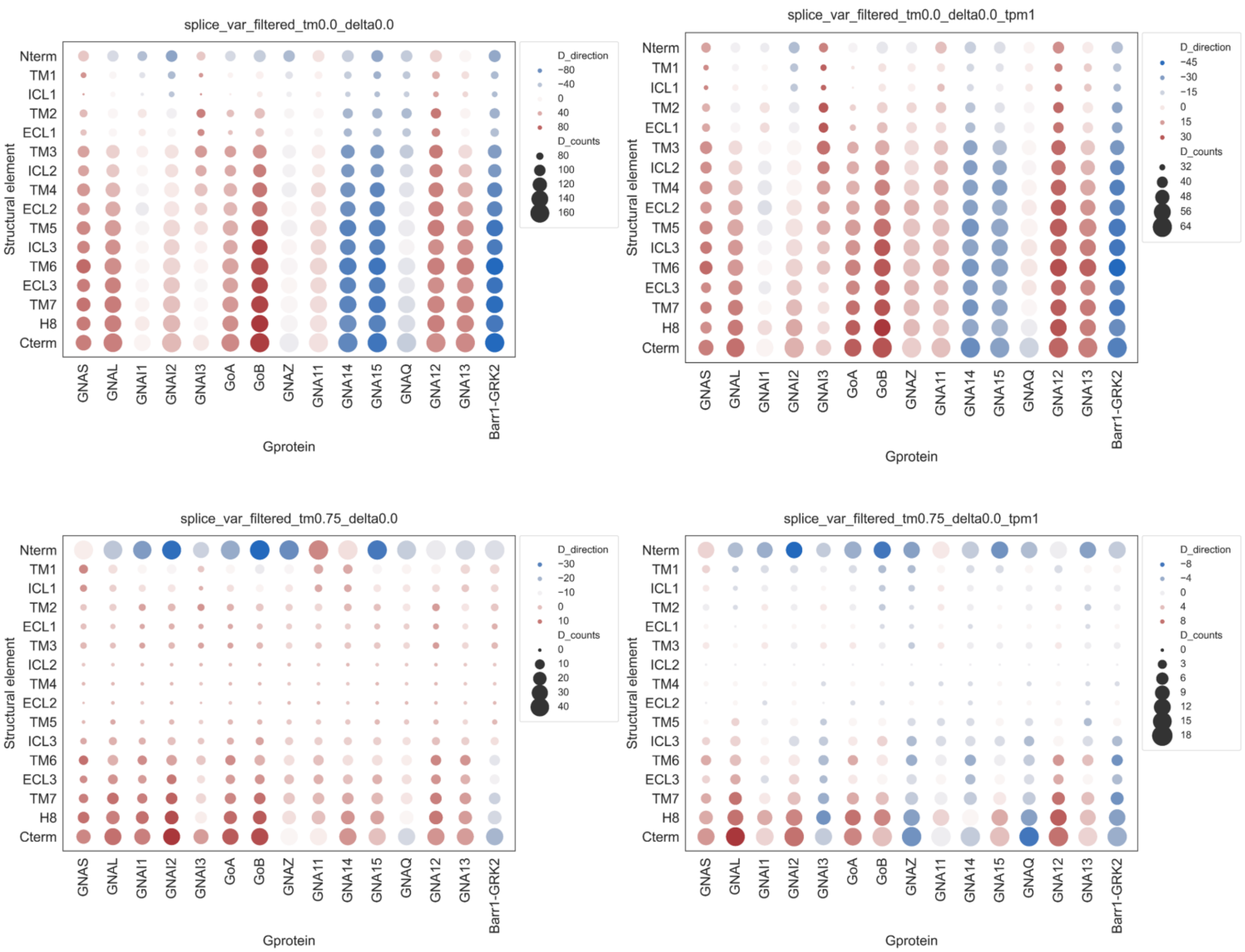
splice_var_filtered_tm0.0_delta0.0
splice_var_filtered_tm0.0_delta0.0_tpm1
splice_var_filtered_tm0.75_delta0.0
splice_var_filtered_tm0.75_delta0.0_tpm1
Structural element
Nterm
TM1
ICL1
TM2
ECL1
TM3
ICL2
TM4
ECL2
TM5
ICL3
TM6
ECL3
TM7
H8
Cterm
Gprotein
GNAS
GNAL
GNAI1
GNAI2
GNAI3
GoA
GoB
GNAZ
GNA11
GNA14
GNA15
GNAQ
GNA12
GNA13
Barr1-GRK2
D_direction
D_counts

Figure S2
